# Analysis of eHealth Search Perspectives Among Female College Students in the Health Professions Using Q Methodology

**DOI:** 10.2196/jmir.1969

**Published:** 2012-04-27

**Authors:** Michael Stellefson, Bruce Hanik, J. Don Chaney, Bethany Tennant

**Affiliations:** ^1^Center for Digital Health and WellnessDepartment of Health Education and BehaviorUniversity of FloridaGainesville, FLUnited States; ^2^Office of Health InformaticsDepartment of Health and KinesiologyTexas A&M UniversityCollege Station, TXUnited States

**Keywords:** eHealth literacy, college students, Q methodology, Internet search, health professionals, medical education

## Abstract

**Background:**

The current “Millennial Generation” of college students majoring in the health professions has unprecedented access to the Internet. Although some research has been initiated among medical professionals to investigate the cognitive basis for health information searches on the Internet, little is known about Internet search practices among health and medical professional students.

**Objective:**

To systematically identify health professional college student perspectives of personal eHealth search practices.

**Methods:**

Q methodology was used to examine subjective perspectives regarding personal eHealth search practices among allied health students majoring in a health education degree program. Thirteen (n = 13) undergraduate students were interviewed about their attitudes and experiences conducting eHealth searches. From the interviews, 36 statements were used in a structured ranking task to identify clusters and determine which specific perceptions of eHealth search practices discriminated students into different groups. Scores on an objective measure of eHealth literacy were used to help categorize participant perspectives.

**Results:**

Q-technique factor analysis of the rankings identified 3 clusters of respondents with differing views on eHealth searches that generally coincided with participants’ objective eHealth literacy scores. The proficient resourceful students (pattern/structure coefficient range 0.56-0.80) described themselves as using multiple resources to obtain eHealth information, as opposed to simply relying on Internet search engines. The intermediate reluctant students (pattern/structure coefficient range 0.75-0.90) reported engaging only Internet search engines to locate eHealth information, citing undeveloped evaluation skills when considering sources of information located on the Internet. Both groups of advanced students reported not knowing how to use Boolean operators to conduct Internet health searches. The basic hubristic students (pattern/structure coefficient range 0.54-0.76) described themselves as independent procrastinators when searching for eHealth information. Interestingly, basic hubristic students represented the only cluster of participants to describe themselves as (1) having received instruction on using the Internet to conduct eHealth searches, and (2) possessing relative confidence when completing a search task.

**Conclusions:**

Subjective perspectives of eHealth search practices differed among students possessing different levels of eHealth literacy. These multiple perspectives present both challenges and opportunities for empowering college students in the health professions to use the Internet to obtain and appraise evidence-based health information using the Internet.

## Introduction

The Internet continues to be widely used to facilitate research and learning for health and medical information. Eight out of 10 Internet users look online for health information, making it the third most popular Web activity next to checking email and using search engines [1]. The pervasiveness of the Internet and the continued evolution of devices that employ Web-based technologies makes obtaining, processing, and understanding health information a critical competency area for medical professionals in training. Among medical professionals around the world, mobile information and communication technologies (eg, smartphones, iPads, and notebook computers) enable frequent Web 2.0 searches for health information [2-5]. Recent studies have highlighted limitations in measuring and evaluating the interchangeable and interrelated skills necessary for information gathering in the highly social Web 2.0 environment [6-8]. The ability to conduct an effective Internet search to locate health information is particularly important for health and medical professional students who represent an especially “plugged in” subgroup of the future public health workforce. Approximately 76% of college students use the Internet frequently for research or homework, while 86% report spending at least some time on social networking sites each week [9]. Given the wealth of health and medical information that exists on the Internet, implementing evidence-based health and medical Internet searches becomes far more complex than simply entering a medical condition or health term into an Internet search engine (eg, Google or Bing) and clicking on the most prominent search result within the selected Web browser.

Obtaining health information using the Internet involves a variety of competencies that health information seekers generally lack [10,11], such as: (1) conducting both basic and advanced information searches; (2) applying Boolean operators to limit Internet search results; (3) differentiating between scholarly documents, authoritative sources, periodicals, and primary versus secondary sources of health information; and (4) comprehending ambiguous eHealth terminology.

Increasingly, health and medical professionals must use at least basic eHealth literacy skills to perform their job-related responsibilities [4,5]. “eHealth literacy” refers to the ability of individuals to seek, understand, and evaluate health information from electronic resources and apply such knowledge to addressing or solving a health problem [12]. The construct reflects the composite of both analytic and context-specific skills that require cognitive-behavioral capabilities to work with technology, critically think about issues of media and science, and navigate through online decision-making resources. The literature has established the need to begin unraveling the basis for cognitive Internet search tasks among medical professionals, especially tasks that may be repeated over long periods of time [4,5,13]. Medical professionals are aware of the need to make evidence-based decisions using eHealth resources [4]; yet, they rarely make evaluative judgments regarding the sources of health and/or medical information they are consuming and habitually visit websites that are perceived to represent high levels of information quality, where cognitive authority is presumed to be high [13].

College students who are professionally trained in the health and medical professions should be taught the knowledge and skills necessary to conduct advanced eHealth information searches on the Internet. These search tasks are complemented by critical appraisals of both the information content and source [14]. The medical education community has recognized the important responsibility of fostering the use of eHealth technologies among future health professionals who will continue to work in the Internet age [15]. Although college students do not encounter the environmental, physical, and resource-related barriers associated with surfing the Internet [8], this population still reports an inability to find desired materials in the digital age [16]. Recent investigations have examined eHealth literacy among college students [14,17]. Stellefson and colleagues [14] conducted a systematic literature review of studies assessing eHealth literacy among college students and found that college students generally lack eHealth literacy skills. Few studies have examined the unique patterns and underlying reasons for college students’ health information search behaviors on the Internet [18], which has led to an incomplete understanding of these tasks. The limited current understanding of eHealth literacy is especially disconcerting when considering the importance of Internet search capabilities among young people studying to become future health and medical professionals.

Hanik and Stellefson [17] attempted to fill the gap in this literature by investigating perceived and actual eHealth literacy among undergraduate health education students studying to become allied health professionals. Participants were asked to complete the Research Readiness Self-Assessment-Health (RRSA-h) [19] online assessment, which measures knowledge/skill sets necessary for performing eHealth searches on specific health and medical topics. The multi-part eHealth search task was operationally defined as: (1) making a determination into possible sources of quality health information; (2) conducting an actual health information search on the Internet; (3) evaluating the quality of the health information retrieved; and (4) answering questions following the analysis of health information that was located and evaluated. A total of 77 undergraduate students (88% female) completed this online assessment and earned subpar actual eHealth literacy test scores (mean overall ability score = 42.6%) [17] as compared to results from a previous study in a similar population [19]. However, it was noted that more advanced students (eg, juniors and seniors) had higher overall eHealth literacy than their younger counterparts did. Although the more senior level students exhibited higher levels of eHealth literacy, it could not be determined whether specific eHealth search attributes were qualitatively different among students possessing high versus low eHealth literacy. It was determined, however, that actual eHealth literacy was markedly inferior to ratings of perceived eHealth literacy [17].

In light of these preliminary research findings, it is important to better understand how personal eHealth search practices are perceived among health and medical professional students. These insights may provide a context for determining the types of characteristics that predict and explain eHealth literacy achievement within this population. The purpose of the current research study was to systematically identify health professional college student perspectives of eHealth search practices. The current study addressed three research questions in hopes of achieving this research aim:

1. How many clusters of health professional college students exist, given information about perceptions of personal eHealth search practices?

2. Which college students belong to the eHealth search clusters that emerge?

3. Which specific perceptions of personal eHealth search practices provide the basis for differentiating the clusters that emerge?

## Methods

To systematically identify health professional college students’ perspectives of their own eHealth search practices, Q methodology [20] was employed. The Q method is a systematic way to study subjectivity and it can be used to reveal various social perspectives that exist on a given topic [20-22]. The Q method fits under the broad umbrella of discourse analysis techniques, which constitutes a large category of research methods that systematically analyzes text-based statements in order to determine underlying patterns or meaning [22]. Within this analysis, individuals are clustered into different typologies based on how they describe themselves [23]. This method of inquiry can provide exceptional insight into how many types of people there are, which people belong to different groups, and which particular variables best differentiate types of people [24]. It also enables researchers to systematically explore a variety of perspectives about an issue to identify important areas that may overlap or differ among unique populations [25]. This methodology has been applied in various disciplines including general nursing research [26], clinical decision making among nurses [27,28], and health care informatics [29].

In Q-method research, participants are the independent variables and the text-based statements they are asked to evaluate are the subject of analysis. Participants are asked to systematically order (or “Q sort”) text-based statements presented to them according to how those statements fit into their own belief system regarding how they believe themselves to be. After participants sort the text-based statements presented to them, the Q method seeks to identify patterns embedded within the Q sorts completed by different participants. Any existing patterns suggest intersubjective orderings of beliefs shared among participants, thus revealing social perspectives [22]. This research technique is valuable because it capitalizes on the strengths of both qualitative and quantitative research [30,31]. For more on the mechanics of Q methodology, the reader is directed to guideline tutorials published within the health and medical research literature [21,26,29].

In the context of the current study, it was hoped that the Q method would help detect any qualitative patterns within undergraduate health professional students asked to consider beliefs about their own personal eHealth search practices. Specifically, the researchers were interested in whether the intersubjective orderings of eHealth search beliefs were common among participants possessing distinct levels of eHealth literacy (eg, basic, intermediate, and proficient). To facilitate this analysis, the Q study protocol was split into three sequential steps: (1) development of the concourse, (2) facilitating the Q sort procedure, and (3) interpreting data from the Q sorts.

### Concourse Development

In Q methodology, a “concourse” is the list of statements that sufficiently represents the “universe of viewpoints” about a topic [32]. To create a concourse of statements made by health professional students regarding attitudes and experiences conducting eHealth searches, a convenience sample of 42 health education majors were recruited from a large research institution in the southwestern United States. Traditional sampling principles and methods used in survey research are not of particular relevance to person sampling in Q methodology; thus, a pragmatic participant selection process was used [29]. Students were asked to respond to a set of statements meant to elicit responses about the students’ personal experiences conducting eHealth searches. All statements were based on cognitive-behavioral constructs posited to be relational within the atomic components of thought theory (ACT) [33], which explains skill development as a process of encoding, strengthening, and proceduralizing declarative and procedural knowledge [34]. Declarative knowledge describes what one knows (eg, facts), whereas procedural knowledge describes whether individuals understand “how to” complete tasks. Complex tasks, such as searching for eHealth information on the Internet, can be described as combinations of declarative and procedural knowledge put to work. The 12 open-ended statements that students were asked to respond to represented combinations of declarative and procedural knowledge necessary for locating and evaluating health information on the Internet.

The 12 statements informed by ACT were written on index cards, color coded, and numbered and each student was given corresponding index cards to write open-ended responses to each statement. For example, each student was asked to respond to the statement, “List the source you use most when you search for health information on the Internet.” After all participants responded to each statement, 504 unique statements (42 students × 12 statements) were generated. Repetitive responses were removed, and a literature review [14] informed the content validity of the 380 statements that were retained for the final concourse. The statements were edited for grammar and readability only to ensure face validity [26] and were grouped together into broad themes that emerged throughout the concourse by way of a constant comparison analysis [35]. To cultivate a greater sense of the most important concepts reported by participants, the number of times each code emerged (across each theme) was quantified to assess saturation within the statement pool. The four overarching identified themes were (frequency of emergent codes corresponding to each theme specified in parenthesis): educational experiences related to conducting eHealth searches (n = 111), confidence in ability to conduct eHealth searches (n = 99), knowledge about conducting eHealth searches (n = 87), and how students engaged the eHealth search process (n = 83).

### Q Sort Procedure

After the final concourse was developed, a subset of 36 representative statements (known as a “Q sample”) was selected to provide a miniature depiction of the larger concourse. This practice is suggested when using Fisher’s experimental design principles in Q methodology [36]. Each statement was randomly assigned a unique numerical identifier from 1 to 36 in order to reduce the probability that participants would recognize conceptually similar statements and cluster related statements together without cognitively processing each statement separately. Table 1 lists the 36 Q statements.

**Table 1:** Statements used for participant Q sorts.

Statement #	Statement content
1	I use Web sources that are easy to cite.
2	I rely on search engines (eg Google, Bing) to find health information for research projects.
3	I have been taught how to find reliable health information on the Internet.
4	I have had assignments that required me to evaluate online health information sources.
5	I use up-to-date information for assignments that require me to find health information online.
6	I use the library databases (eg, EBSCO or CSA) when I search for information.
7	I seek help from library staff for difficult Web searches.
8	I get feedback from professors regarding the quality of Web resources I use for homework assignments.
9	I check the ending of Web addresses (eg, .com, .gov, or .edu) when I search for information online.
10	I consider the source of online information when I find useful information for my research projects.
11	I usually have at least one assignment per semester that requires me to conduct a Web search for health information.
12	I brainstorm to help figure out the health information that is important for my project.
13	I know how to critically evaluate online health information sources.
14	I evaluate online health information that I use for projects such as research assignments.
15	I finish research projects, such as papers, at least one week before their due dates.
16	I look for up-to-date online health information when I conduct Web searches.
17	I go to the library when I start a research project.
18	I can figure out how to find information that is unfamiliar to me.
19	I know where to find reliable online health information.
20	When I am assigned to complete a research paper, I do not hand in the first draft as the final product.
21	I use search engines (eg, Google or Bing) when I search for health information online.
22	I get flustered looking for health information I know little or nothing about.
23	I find it difficult understanding new health information found on the Internet.
24	I do not know where to find reliable online health information.
25	I know how to use Boolean operators.
26	I know what is meant by “peer review.”
27	I am confident in my ability to find reliable health information online.
28	I use health information that I can easily understand.
29	I know what Boolean operators are.
30	I have difficulty finding information when I use library databases such as EBSCO or CSA.
31	I evaluate health information sources when conducting health information searches on the Internet.
32	I know what a primary source is.
33	I go to my professor for help to make sure I use quality health information for research projects.
34	I follow references back to the original source when I find online research studies/reports that are useful for research assignments.
35	I can find useful sources of health information using the library database.
36	I use refined search parameters to narrow my online health information searches.

**Figure 1:**
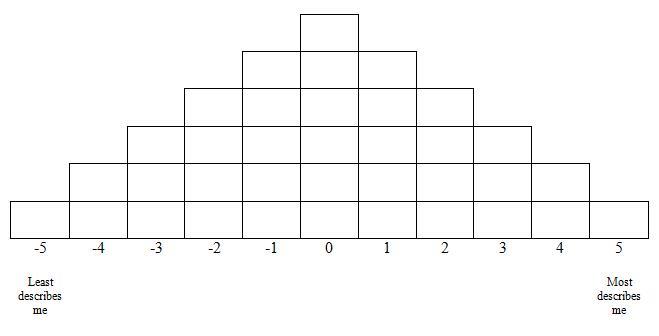
Final distribution of Q sort procedure (Q sort table).

These representative statements were then rank-ordered by the study participants in what is known as a Q sort task. To complete the Q sort, participants were instructed to order the statements according to which statements described them the most and which described them the least when considering their attitudes and experiences conducting eHealth searches. This encouraged participants to sort the cards such that the completed sort would have the shape of a triangle. Columns at both extremes of the triangle possess one card and each column incrementally closer to the center possesses an additional card, with the middlemost column containing 6 cards (thus resembling a quasi-normal distribution). Each participant’s Q sort consisted of 11 columns. The leftmost column was assigned a score of –5 (least descriptive) and the rightmost column was assigned a score of +5 (most descriptive). Figure 1 provides a visual illustration of the quasi-normal distribution of each participant’s Q sort.

In order to make the overwhelming task of rank-ordering 36 statements more manageable, participants were instructed to read all the statements first to get an impression of the range of statements they were asked to evaluate, and then they were asked to sort the cards into 3 distinct piles: one pile for statement cards that described them the least, one pile for cards that did not describe them at all, and one pile for statement cards that described them the most. Participants were then told to take the cards that were least descriptive and order them according to the pattern depicted in Figure 1, beginning at the leftmost side of the distribution pattern. Once the least descriptive index cards had been sequentially laid out, the same procedure was initiated from the right side of the triangle, this time beginning with the most descriptive index cards. The cards in the neutral pile were assigned to the remaining positions that were left vacant in the middle of the distribution after plotting the least and most descriptive statements. Each participant received instructions for completing this task and completed their individual Q sorts in a room by themselves with no help from others.

Once the ranking task was completed, each card was assigned a score based on the column position it occupied (see Figure 1). For example, if a student assigned Index Card 23 to the second column (from the left), that card would be assigned a score of –4, which would reflect that the student believed she had little difficulty when attempting to understand new health information found on the Internet. The authors entered each participant’s rating for each card into a data matrix for analysis.

### Participants 

To recruit participants to complete the Q sort procedure, personalized emails were sent to a convenience sample of 20 undergraduate health education students who had recently participated in the aforementioned study assessing eHealth literacy among college students [17]. The recruitment email requested participation in what was described as a follow-up study. Emails were sent weekly over the course of approximately one month to solicit participation. Participants were eligible to receive US $10 for participation. The recruitment goal was to secure participation from 18 individuals because the number of participants in a Q study should be less than or equal to one-half the number of Q statements [23]. In Q-method research, the number of participants is not necessarily important; rather, it is important that there exists representation of different viewpoints about the theme of the study [37]. Eligible participants were asked to provide relevant biographical and demographic information upon enrollment in the Q study, such as major, emphasis area, grade point average (GPA), and age.

In Q-method research, factor interpretation frequently involves considering relevant independent variables to determine characteristics that may be shared among clusters of participants [25]. Thus, to further aid in interpreting and defining factors, Q study participants completed the RRSA-h before completing the Q sort, and their scores were recorded [17]. The RRSA-h consists of multiple choice knowledge questions and skill-based problems that generate an overall actual ability score (score range 0-23). Within the current investigation, the research team used the overall scores from administration of this assessment (mean 18.46 points, SD 4.11) to categorize Q study participants as having basic, intermediate, or proficient eHealth literacy. Students in the basic group were defined as those students scoring at or below the 25th percentile of all scores (ie, ≤ 16 points); the intermediate group was defined as those students scoring within the interquartile range (ie, 17-19 points), and the proficient group was defined as those students scoring at or above the 75th percentile (ie, ≥ 20 points).

### Data Analysis

Data from participants’ Q sorts of the 36 statements were analyzed using Q-technique exploratory factor analysis (EFA) [21]. This analytical technique clusters individuals into “types” and provides quantitative data that describes the similarities of participants using correlations between the individual Q sorts completed by participants [23,29,38]. Whereas “R-technique” EFA [39] typically analyzes a two-dimensional data matrix where the rows are defined by participants and the columns are defined by variables, Q-technique factor analysis considers a two-dimensional data matrix where the rows are defined by the statements sorted by the participants and the columns are defined by the participants themselves. Similar Q sorts that correlate significantly with each other form a group, and each factor represents a group of individuals that share similar views and experiences related to the study theme [25]. Q-technique factor analysis produces a weighted, or synthetic, Q sort for each rotated factor by using a weighted averaging method to calculate the score for each statement for each factor [32]. The final data obtained from the individual Q sorts were entered into SPSS version 17.0, and Q-technique EFA using principal components analysis with varimax rotation was used to identify patterns among the Q sorts to determine which participants were correlated with factors retained in the analysis. To determine the correct number of factors to retain for analysis, bootstrap factor analysis was used concomitantly with the eigenvalues-greater-than-one rule [40,41].

The final step in the analysis involved an effort to determine which (if any) of the 36 statements provided a basis for differentiating the clusters of students identified (ie, the factors). Factor scores were computed for each statement and plotted for each retained factor to determine the extent to which each cluster of students agreed or disagreed with how descriptive each statement was regarding their own perspectives on conducting eHealth searches. Factor scores less than –1.0 and more than +1.0 were more than one standard deviation from the factor score mean, so these were the statements of least or most agreement among the individuals defining the factors [24]. Negative factor scores indicated that participants making up the cluster did not agree that the statement described their attitudes or experiences conducting eHealth searches. Positive factor scores indicated that participants did agree that the statement described their perspective on conducting eHealth searches.

## Results

A total of 13 students agreed to participate in the Q study following recruitment (response rate = 65%). All participants were female and the majority (8/13, 77%) were third- and fourth-year students (ie, juniors and seniors) majoring in health education with an emphasis in the allied health professions. This number of participants was judged to be sufficient given that Q-method research requires the number of participants be small relative to the number of ranked variables [22,23]. The students possessed an average GPA of 3.26 (SD 0.42) points on a 4-point scale and were categorized as having either basic (n = 4), intermediate (n = 5), or proficient (n = 4) eHealth literacy based on their performance on the RRSA-h. The mean RRSA-h score was 18.46 (SD 4.11) out of a maximum of 23. The scores derived from the administration of the RRSA-h demonstrated adequate internal reliability (alpha = .78). Table 2 describes relevant Q study participant characteristics.

**Table 2:** Demographic characteristics of participants.

Characteristic		n (%)
**Sex**		
	Female	13 (100)
**Student classification**		
	Freshman	1 (8)
	Sophomore	4 (31)
	Junior	2 (15)
	Senior	6 (46)
**Degree track**		
	Allied health	12 (92)
	Community health	1 (8)

The Q-technique EFA of the 36 statements yielded 3 factors representing 3 salient perspectives among study participants. The 3-factor structure suggests there were 3 types of health education students with regard to eHealth search practices. The varimax-rotated factor pattern coefficients (ie, the correlations between each participant with each of the 3 factors) suggested that the 3 factors were reasonably independent of one another. Table 3 describes the varimax-rotated component matrix that lists the pattern/structure coefficients for each participant on all three retained factors. Every participant had at least one pattern/structure coefficient on one factor that was at least equal to 0.5, which indicates that each participant was moderately correlated with at least one retained factor [42].

**Table 3:** Factor pattern/structure coefficients for participants.

Participant^a^	Proficient resourceful	Intermediate reluctant	Basic hubristic
P1	0.56^b^	0.24	0.46
P2	0.67^b^	0.12	0.25
P3	0.73^b^	0.26	0.47
P4	0.80^b^	0.31	-0.26
I3	0.71^b^	0.24	0.36
I4	0.78^b^	-0.01	0.38
I5	0.60^b^	0.35	0.36
I1	0.11	0.89^b^	0.09
I2	0.34	0.85^b^	0.05
B3	0.20	0.75^b^	0.39
B1	0.23	0.19	0.54^b^
B2	0.08	0.40	0.74^b^
B4	0.37	-0.13	0.76^b^

^a ^P = proficient group, I = intermediate group, B = basic group

^b ^Pattern/structure coefficients above 0.50

The first factor was correlated with all participants who were proficient achievers on the RRSA-h and with3 participants who were intermediate achievers. The second factor was highly correlated with two participants from the intermediate group and one participant from the basic group. The third factor was correlated with the 3 remaining participants in the basic group. Thus, after analyzing quantitative performance on the RRSA-h in relation to findings from the Q-technique EFA, it was determined that perspectives of personal eHealth search practices did, in fact, differ among health professional students of basic, intermediate, and proficient eHealth literacy. More than half of the students (7/13, 54%) clustered on the proficient factor, while 3 students clustered on each of the 2 other factors. The authors determined that the magnitude of the differences between the primary and cross loadings for each participant across each factor were large enough (≥ 10% difference) to consider each participant as a defining individual for the factor with their largest pattern/structure coefficient.

The factor scores [38] of each of the 36 statements rated by the Q participants were used to determine which eHealth search practices were associated the most and the least with each of the three types of students. In the lexicon of Q methodology, these statements are called “distinguishing statements” because they help to explain the uniqueness of each factor [25]. Table 4 presents the statement factor scores greater than 1.0 or less than –1.0. These statements informed the specific differences in eHealth search perspectives among participants possessing different levels of objective eHealth literacy, and also provided a deeper understanding of the opinions that discriminated the three clusters of students. Moreover, the factor scores were used to further represent the characteristics of each cluster, with the first factor describing proficient resourceful students; the second factor describing intermediate reluctant students; and the third factor describing basic hubristic students.

**Table 4:** Salient statements for retained factors less than –1 and greater than +1.^a^

Statement #	Proficient resourceful		Intermediate reluctant	Basic hubristic
2	-1.96	1.34		
3				1.99
6	1.85			
7	1.12	-1.38		-1.11
8		-1.07		
9		1.37		
11				1.21
12	1.01			-1.50
13		-1.23		
15				-1.64
16	1.15			
17		-1.37		
18				1.24
19				1.83
20	1.53	2.04		-1.79
21	-1.09	2.31		
22	-1.49			
23				-1.08
24	-1.25			-1.04
25	-1.66	-1.56		
26				1.62
27				1.64
28	-1.29	1.87		
29	-1.75	-1.52		
32	1.28			
34		-1.16		

^a ^Factor scores between –1.0 and +1.0 were removed from table

### Proficient Resourceful and Intermediate Reluctant Students

The proficient resourceful students (PRS) described themselves as relying on multiple resources to obtain eHealth information (Statements 2, 6, and 12), as opposed to simply relying on Internet search engines to conduct Web searches (Statements 2 and 21). They also indicated that they worked well with research partners (including library staff members) to brainstorm ideas for research projects and seek further assistance to conduct difficult Internet searches (Statements 7 and 12). Conversely, intermediate reluctant students (IRS) reported relying solely on Internet search engines when conducting eHealth searches (Statements 2 and 21). The IRS group also described themselves as working more independently with less reliance on using library resources or instructors to obtain assistance when searching (Statements 7, 8, and 17).

The PRS group described being able to search for up-to-date, even unfamiliar, health information on the Internet (Statements 16, 22, and 24), whereas IRS tended to limit the breadth of their eHealth searches, tending not to seek out original documents from the reference sections of books and manuscripts (Statement 34). Furthermore, IRS perceived themselves as lacking critical skills for evaluating sources of eHealth information (Statement 13). Both types of students reported not knowing what Boolean operators were or how to use them to effectuate eHealth searches (Statements 25 and 29).

### Basic Hubristic Students

Basic hubristic students (BHS), like their IRS counterparts, preferred to search for eHealth information independently (Statements 7 and 12). They also reported being procrastinators who were more likely to identify with submitting a first draft as a final research product (Statements 15 and 20). However, BHS were the only participants to strongly identify with (1) receiving some instruction and practical experience conducting health research on the Internet (Statements 3 and 11), and (2) possessing confidence when attempting to locate and recognize reliable eHealth information, even when researching an unfamiliar topic (Statements 18, 19, 23, 24, 26, and 27).

## Discussion

Q methodology was chosen as a robust qualitative technique to measure the subjective perspectives of eHealth search practices among undergraduate students enrolled in a health professional degree program. This study applied a Q sort technique to identify clusters of students representing different levels of eHealth literacy. Each cluster was described in terms of perceived skill level, confidence in ability to conduct eHealth searches, and past educational experiences. Three distinct viewpoints were revealed concerning perceptions of eHealth search practices among different “types” of students. These three viewpoints were found to share some common elements, especially when considering participants’ personal eHealth literacy (ie, basic, intermediate, or proficient). In addition, the specific similarities and differences between student clusters are useful to consult when determining which component eHealth search skills are typical among different types of undergraduate health professional students. The following discussion makes use of the distinctive statements identified above to shed light on how results from this Q-method study might be used to suggest implications for the instruction of different “types” of college students majoring in the health professions.

### Proficient Resourceful and Intermediate Reluctant Students

The PRS described themselves as students who relied on multiple resources to obtain eHealth information, as opposed to simply relying on Internet search engines. They worked well with library personnel to brainstorm ideas for research projects and sought guidance on how to conduct difficult searches. A previous study on eHealth search tendencies among college students noted that students are apt to seek digital health information from multiple, complementary sources of information [43]. Health professional students may benefit from being made aware of library support services within college and university settings that can be utilized to strengthen information literacy competencies necessary to ensure students know (1) how eHealth information is organized, (2) where to find reputable sources of health information on the Internet, and (3) how eHealth information should be used in practice. For example, it has been noted elsewhere that university librarians, along with the resources they can provide, play a critical role in providing insights and guidelines for health information literacy and Internet search skills [18].

The IRS were more reliant on Internet search engines to conduct eHealth searches. College students have reported using Internet search engines almost exclusively to locate online health information [18,44,45]. Research has also shown that college students resort to using rudimentary retrieval methods (eg, the use of unrefined search terms or selecting only the most apparent and visible search results) to gather online health information [44,46]. This study is the first to report that even moderately eHealth-literate students identify with only using search engines when conducting eHealth searches. To support continued eHealth literacy development, training programs in computer literacy can increase both absolute and relative access to eHealth resources by teaching students to make use of new and existing technological resources (ie, library research databases, RSS feeds, Twitter, and Facebook). These accessible, information-seeking technologies can augment students’ general exploration using Internet search engines. Additionally, general social media skills should become extended into training programs designed to provide instruction in eHealth literacy [47]. As part of the movement towards Medicine 2.0 [48], it has been suggested that dimensions of social media, such as synthesizing professional and non-professional advice and using apomediaries (ie, expert sources) to filter relevant and trustworthy information, be included within eHealth literacy interventions. Instruction in these additive content areas will help ensure that health professional students are able to locate, evaluate, and use eHealth information at a level greater than the general population.

The IRS also perceived they lacked evaluation skills when considering sources of eHealth information. Previous work has shown perceived ability to evaluate eHealth information to correspond with actual evaluation ability among college students in the health professions [17]. Search protocols and criteria for evaluating eHealth information are often implied and not explicitly understood by health professional college students; thus, they resort to simply trusting search engine results [18,49] and/or relying on the website sponsor, appearance, or other non-content–related cues to form credibility judgments [50]. Even intermediate eHealth-literate college students may recognize personal limitations evaluating eHealth information, which indicates the need for explicit criteria that students can reference when evaluating eHealth information in a multifaceted, complex Web environment. These guidelines should support health and scientific literacy by enabling students to perform literary and numerical tasks necessary to comprehend and respond to health care information that is provided in an often- convoluted Web context. Standards should clearly articulate the importance of systematically evaluating sources of eHealth information to verify reputability, ensure validity of information, and promote understanding [18].

Interestingly, neither PRS nor IRS reported receiving formalized training on how to search for quality health information on the Internet. There are a variety of competencies associated with obtaining eHealth information, including the knowledge, skills, abilities, and other attributes (KSAOs) necessary to conduct basic and advanced information searches, apply Boolean operators to limit search results, and understand (sometimes ambiguous) eHealth terminology. These KSAOs may be limited, even among high-performing students. Previous research has shown that college students are not equally capable of accessing health information online [43]; therefore, to ensure that students of all eHealth literacy levels (even proficient levels) are appropriately searching for eHealth resources, it is important that multidisciplinary training programs be integrated within allied health and medical education programs to deliver instruction that will provide health professional students with experiences to further develop KSAOs in each facet of eHealth literacy. While such KSAOs may be overlooked when training students who have aspirations of gaining employment in patient care settings (eg, nursing, medical assistants, physical therapy, and occupational therapy), students living in the Internet age will likely need to be prepared to use search guidelines in the clinical and community environment to assist in finding preventive, diagnostic, and treatment information.

Navigating through health information obtained on a mobile device can present the user with unique Internet search and retrieval obstacles that are separate and distinct from searches of the Internet on a desktop CPU. Novel coursework in media literacy can assist in training health professional students living in the digital age to access and use health information available in the new age of mHealth applications. More practical continuing education and learning experiences should be provided to both instructors and students alike to ensure that mobile and digital technologies are included as a subtopic of eHealth literacy. It is important that attention is given to supporting instructional programs using mHealth applications within public health interventions.

### Basic Hubristic Students

The BHS, like the IRS, preferred to work independently when searching for eHealth information. Students who possess inferior skills searching for and evaluating eHealth information should be encouraged to seek out consultation during the eHealth information-seeking process. Other research indicates that simply observing the thought processes and search tendencies of higher-level students could indirectly result in better learning outcomes [51]. This perspective suggests that modeling effective skills, abilities, and other attributes (SAOs), such as task adherence, may help to enhance searches completed by lower-performing students who may be negatively affected by tendencies toward procrastination. Users’ affective states and social context affecting information needs are relevant variables that may influence perceived search task difficulty, search effort, and success [18]. By delaying the search for quality eHealth information, students may tend to resort to using elementary Internet search methods, such as using general search terms within basic Internet search engines. Procrastination may also limit the effort that students can devote to using more scholarly sources of health information from institutional eLibrary databases. Future research should determine the particular cognitive and personality characteristics that predict and explain eHealth literacy outcomes among health professional students.

The BHS were the only participants to describe themselves as receiving instruction on how to conduct Internet searches for health information. Furthermore, the BHS described themselves as possessing confidence in their ability to find and recognize reliable eHealth information even when researching an unfamiliar topic. This is one major distinguishing characteristic that separated low performers from the more advanced student clusters. Speculation in previous research [14,17,19,52] suggests that low-performing college students may have an inflated sense of their eHealth literacy skills. While college students majoring in the health professions may believe they possess the necessary KSAOs to effectively engage in eHealth searches on the Internet, this belief of personal capability may not be substantiated when considering evidence of their eHealth “illiteracy” (eg, proficient and intermediate performers unaware of what Boolean operators were). A common phenomenon in engagement with health and research based information is a sense of overconfidence in the skills required to understand and utilize data [53]. To investigate whether this phenomenon is relevant to the current line of inquiry, the continued measurement of self-efficacy for conducting eHealth searches should help determine how perceived eHealth search behaviors map onto actual behaviors among a variety of health professional college students.

Table 5 provides definitions of the dimensions of eHealth literacy [12] discussed above plus practical application examples that can be used in the training of health professional students. College students preparing to become medical and allied health professionals should obtain customized eHealth literacy training in areas where competency deficiencies are present.

**Table 5:** Practical applications for training allied health students using the dimensions of eHealth literacy.

Dimension	Definition	Practical applications for training
Media	Skills to apply cognitive process and critical thinking to media messages	Provide opportunities for students to gather and assess health information from a variety of media sources.
		The authors suggest instructors of courses utilize the media literacy lesson plans created as part of student reporting labs at PBS (http://www.studentreportinglabs.com/lesson-plans).
Information	Skills to know where to go to find the appropriate information and how to use the information once collected	Information literacy skills should be incorporated very early into the curricula. The authors suggest readers review Kingsley et al [54,55] and Levine et al [56] for case studies on incorporating information literacy skills in an allied health curriculum.
Computer	Skills to be able to use computers to solve problems	Provide online or hybrid computer literacy training that requires students to become more comfortable with using computers.
		Provide assignments that require allied health students to conduct Internet searches and validate the information found.
Scientific	Skills to understand the political and sociological dimensions of science	Require a research-training component as part of all allied health degree programs.
Health	Skills to understand health information and make appropriate health decisions	Incorporate training within allied health classes on how students use valid and reliable health information from sources to make health decisions.
		Incorporate the free, online Health Resources and Services Administration (HRSA) training (http://www.hrsa.gov/publichealth/healthliteracy/index.html).

### Limitations

Participation in the current study was limited to a convenience sample of female respondents. It is important to note that this limitation was reflective of the disproportionate number of female students enrolled in this particular health education major, and the literature has indicated that female students are more likely than male students to use the Internet to locate health information [43]. Nevertheless, future studies would benefit from using multiple demographic and psychographic variables to inform participant sampling designs for the purpose of uncovering more descriptive differences in self-reported perspectives.

There was also an uneven number of first- and second-year students versus third- and fourth-year students. This potentially skewed the results considering that the more senior students likely possessed more experience searching for health information on the Internet. Because of the small, nonrandom samples characteristic in Q methodology, findings may not be broadly applicable to various groups of undergraduate health professionals. The current Q study can only be considered representative of the continuum of perspectives that may exist about eHealth search practices among undergraduate female health education students attending a large, research-oriented university. Eliciting subjective perspectives of personal eHealth search practices at multiple types of college and universities, representing schools of diverse backgrounds (eg, different research and teaching institutions) with varied allied health and medical specialty areas (eg, nursing, physical therapy, public health, and physician assistants), may very well result in different perspectives emerging. To fully develop population validity for the variety of students in the health and medical professions, future studies should examine the link between health profession major area of emphasis and perceptions of eHealth search practices.

Although the Q technique has strengths, such as enabling comparisons across subjective topics [19,57], it is possible that other perspectives do exist and were not reflected in the factor structure reported in the current study. The meaning (and naming) ascribed to each of the three retained factors was contrived by the research team on the basis of results from previous research [17]; this could represent research bias that is a threat to the internal validity of Q studies [58]. In future replications of this research design, it would be useful to assess the inter-rater reliability of the number of factors retained, and especially how retained factors are described by different raters. Also, any number of participants in this study may have misunderstood what could have been perceived as complex instructions for the Q sort, which could have led students to misrepresent their perspectives on eHealth search practices. Future studies should confirm whether or not students clearly understood the instructions for the Q sorting procedure.

As well, study participants had already completed the RRSA-h assessment and also received feedback on their performance before completing their individual Q sorts. This represented a testing threat to the internal validity of results from this study because feedback on the RRSA-h may have altered students’ perceptions of personal eHealth search skills along with their perceived need for instruction and assistance when searching for eHealth information. Finally, the reliability of the Q sorts could not be verified by a test–retest procedure due to time limitations inherent within the research protocol.

### Conclusion

The context where eHealth literacy is applied and understood is dynamic and evolving [47]. Thus, the need for instructional eHealth literacy programs has received [18,44], and should continue to receive, widespread support from the public health and medical education communities. The findings of this study suggest a careful analysis of multiple perspectives that exist related to eHealth search practices among female health professional college students. Results offer an important context for deepening knowledge about the dynamics of participation and empowerment related to eHealth literacy in this population. Previous research has shown college students to have varying degrees of eHealth literacy [14,17]. This study is among the first to reveal multiple, practically significant student perspectives on eHealth search skills and behaviors. It also helped to begin the discourse conceptualizing the different ways that eHealth search practices may differ when considering the KSAOs students may or may not possess. The subjective perspectives of participants clustered across three relatively distinct factors that coincided with participant performance on a valid and reliable eHealth literacy assessment. The characteristics describing each of these cluster sets (ie, basic hubristic, intermediate reluctant, and proficient resourceful) provide future eHealth literacy researchers with opportunities to test interesting hypotheses that can further explain achievement traits among different types of health and medical professional students. With more instruction and coursework specifically devoted to addressing the rapid shifts in the informational landscape created by Web 2.0 tools and environments, it is likely that any chasms existing between upper- and lower-performing health professional students will be reduced. Moreover, there will a greater proportion of undergraduate students in the allied health and medical professions who will become proficient when using the Internet to locate and evaluate eHealth information.

## Abbreviations

ACT: atomic components of thought theory
BHS: basic hubristic students
EFA: exploratory factor analysis
GPA: grade point average
IRS: intermediate reluctant students
KSAOs: knowledge, skills, abilities, and other attributes
PRS: proficient resourceful students
RRSA-h: Research Readiness Self-Assessment-Health
SAO: skills, abilities, and other attributes
